# Effect of Heat Treatment on Repetitively Scanned SLM NiTi Shape Memory Alloy

**DOI:** 10.3390/ma12010077

**Published:** 2018-12-26

**Authors:** Zhong Xun Khoo, Jia An, Chee Kai Chua, Yu Fang Shen, Che Nan Kuo, Yong Liu

**Affiliations:** 1 Institute for Sports Research, School of Mechanical and Aerospace Engineering, Nanyang Technological University, Singapore 639798, Singapore; zkhoo001@e.ntu.edu.sg; 2 Singapore Centre for 3D Printing, School of Mechanical and Aerospace Engineering, Nanyang Technological University, Singapore 639798, Singapore; anjia@ntu.edu.sg (J.A.); mckchua@ntu.edu.sg (C.K.C.); 3 Department of Bioinformatics and Medical Engineering, Asia University, Taichung 41354, Taiwan; cherryuf@gmail.com; 4 3D Printing Medical Research Institute, Asia University, Taichung 41354, Taiwan; 5 School of Mechanical and Aerospace Engineering, Nanyang Technological University, Singapore 639798, Singapore; yliu.email@yahoo.com

**Keywords:** 3D printing, 4D printing, additive manufacturing, Selective Laser Melting, NiTi, shape memory alloy

## Abstract

Selective Laser Melting (SLM) has been implemented to address the difficulties in manufacturing complex nickel titanium (NiTi) structures. However, the SLM production of NiTi is much more challenging than the fabrication of conventional metals. Other than the need to have a high density that leads to excellent mechanical properties, strict chemical compositional control is required as well for the SLM NiTi parts to exhibit desirable phase transformation characteristics. In addition, acquiring a high transformation strain from the produced specimens is another challenging task. In the prior research, a new approach—repetitive scanning—was implemented to achieve these objectives. The repetitively scanned samples demonstrated an average of 4.61% transformation strain when subjected to the tensile test. Nevertheless, there is still room for improvement as the conventionally-produced NiTi can exhibit a transformation strain of about 6%. Hence, post-process heat treatment was introduced to improve the shape memory properties of the samples. The results showed an improvement when the samples were heat treated at a temperature of 400 °C for a period of 5 min. The enhancement in the shape memory behavior of the repetitively scanned samples was mainly attributed to the formation of fine Ni_4_Ti_3_ metastable precipitates.

## 1. Introduction

Shape memory alloys (SMAs) have been widely utilized as a result of their excellent functional properties and high magnitude of actuation energy density [1,2,3,4]. Among the different SMAs, nickel titanium (NiTi) has the best combination of properties including a high percentage of shape recovery, recovery stress and superelastic strain [4,5,6,7,8]. However, the limitations of the traditional manufacturing technologies and the poor machinability of NiTi have critically restricted its full potential applicability [2,9,10,11,12,13]. Nevertheless, the fabrication mechanism of additive manufacturing, in particular, Selective Laser Melting (SLM), has provided a means to address the issues encountered in the production of complex NiTi smart structures [14,15,16].

In recent years, owing to the trend of using shape memory materials for 4D printing applications [14,15,16,17], a number of research on SLM manufacturing of NiTi were performed to study the various aspects of SLM-produced NiTi [1,3,9,11,18,19,20,21,22,23,24,25,26,27,28,29,30,31,32,33]. When compared to the fabrication of conventional metals via the SLM process such as titanium alloy [34], the production of NiTi is much more challenging. Other than requiring the SLM parts to exhibit high density that results in good mechanical properties, strict compositional control is also needed for the SLM NiTi to possess suitable phase transformation characteristics. Additionally, obtaining a high percentage of shape recovery strain from the SLM NiTi specimens is another challenging task that requires the understanding of the processing-microstructure-property relation.

The shape memory effect and superelasticity demonstrated by NiTi SMA is due to the reversibility of its phase transformation from martensite to austenite and vice versa. However, the introduction of plastic deformation such as slip and dislocation are irreversible and the strains generated could not be restored even upon heating [35,36]. Moreover, it has been reported that the formation of dislocations in the NiTi material could be initiated at a low-stress level of about 100 MPa [37]. Hence, it is essential to increase the critical stress level that the plastic deformation occurs in order for the NiTi to exhibit good shape memory properties.

In physical metallurgy, there are four main methods to increase the critical stress magnitude: alloy-hardening, work-hardening, precipitation hardening and grain refinement [2,30,35,36,38,39]. However, alloy-hardening is not preferred as it would alter the transformation temperatures. Furthermore, unlike the conventional way of producing NiTi parts, the SLM process has provided the freedom of fabricating structures with complex geometries. Thus, it is not necessary to deform or cold-work the finished components into the desired shapes. In addition, recrystallisation is required for the work-hardened components to restore back to the defect-free lattice, which could further increase the production time. Hence, heat treatment could be introduced to possibly generate the other two types of strengthening mechanism: precipitation hardening and grain refinement.

Nevertheless, SLM was only recently adopted for the fabrication of NiTi parts. The effects of heat treatment on SLM NiTi samples have not been fully understood. Lately, some researchers have explored the influences of heat treatment on additive manufactured NiTi parts. For instance, Saedi et al. presented the effects of ageing on the shape memory properties of solutionised Ni-rich SLM NiTi samples [30]. They found out that samples aged at 350 °C for 18 h had the highest transformation strain and the lowest permanent strain. As compared to the non-heat treated samples, the improvement in the superelasticity was associated with the precipitation hardening effect as a result of Ni_4_Ti_3_ precipitate formation. However, lower transformation strains were observed in the 450 °C aged samples. They attributed it to the decrease in the volume fraction of Ni_4_Ti_3_ precipitates, resulting in the lowering of hardening effect and strength of the samples. Meanwhile, Marattukalam et al. utilized Laser Engineered Net Shaping process to fabricate their NiTi samples [40]. They observed different morphologies of martensite formed with various heat treatment conditions. A higher volume fraction of martensite was also found on the heat treated samples than the non-heat treated samples. Moreover, the grain size of the samples was noticed to have increased with the implementation and temperature of heat treatment. They have also detected an increase in the transformation strain with samples heat treated at 500 °C due to the increase in the volume fraction of the martensitic phase. However, the strain recovery decreased when the heat treatment temperature increased to 1000 °C as a result of the stress relief effect.

Nonetheless, both research groups have presented different optimal heat treatment temperatures to induce the desired shape memory responses. This may be caused by the different additive manufacturing technologies used, processing methodologies and sample preparations, etc. More studies are needed to determine a suitable heat treatment temperature for the SLM NiTi to demonstrate a higher transformation strain. Furthermore, in the prior work, a new approach—repetitive scanning—was implemented to improve the transformation strain as compared to the conventional single scanned SLM NiTi samples [31]. A promising result of an average of 4.61% transformation strain was obtained by characterizing the repetitively scanned samples under tensile mode. However, there is still room for improvement since the conventionally-produced NiTi can exhibit a transformation strain of about 6% [10]. Therefore, the objective of this paper is to enhance the shape memory properties of repetitively scanned SLM NiTi samples through the implementation of post-process heat treatment.

## 2. Experimental Procedure

### 2.1. Material

**Figure 1:**
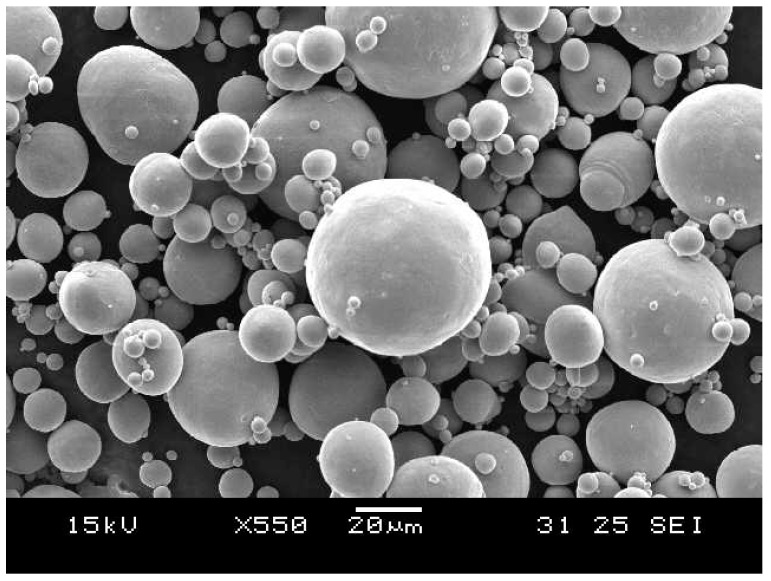
Micrograph of the NiTi powder used.

Figure 1 shows the pre-alloyed NiTi powder (Metal Powders and Materials LLP) used for the fabrication of repetitively scanned NiTi samples. The powder size ranges mainly from 20 to 50 μm. The average chemical composition of the powder was determined by two methods: (1) by utilizing the empirical relationship between the chemical composition of NiTi and its martensitic transformation start (M_s_) temperature [41]; and (2) by performing the energy-dispersive X-ray spectroscopy (EDX) (Oxford Instruments, Inca x-stream, Oxfordshire, UK) testing on the NiTi powder. The phase transformation temperatures (M_s_ and martensitic transformation finish (M_f_) temperatures, austenitic transformation start (A_s_) and finish (A_f_) temperatures) of the NiTi material were determined by conducting differential scanning calorimetry (DSC) testing. Meanwhile, the EDX result is shown in Table 1. The average atomic percentage of the NiTi powder is approximated to be 50.16% Ni and 49.84% Ti.

### 2.2. Material Processing

Repetitively scanned SLM NiTi samples were produced by using a customized SLM equipment (Precision Laser Solutions, Singapore) under an argon (purity more than 99.9995%) atmosphere. The volume of oxygen within the fabrication chamber was maintained below 0.1%. A thick layer of powder was first deposited on to an aluminum building platform, followed by directing the laser beam to the powder bed. However, only the upper layer would undergo melting and solidification, leaving a layer of powder separating the samples and platform. This method of production minimizes contamination due to the reaction of NiTi with aluminum. Moreover, it allows easy and convenient removal of the samples.

During the fabrication process, the NiTi powder was subjected to two-step scanning. Laser powers of 25 and 60 W were selected for the first and second scan, respectively. The laser scanning speed was kept constant at 3600 mm/s throughout. These parameters were determined during the optimization process mentioned in the prior work [31]. The relative density of the samples was measured using the buoyancy method and has an average value of 96.4% [42,43].

To determine the various properties, samples with dimensions of 5 mm by 10 mm and 5 mm by 78 mm were fabricated. The longer strip samples (5 mm by 78 mm) were then wire cut to a width of about 3.5 mm to remove the uneven sides due to powder adhesion. Subsequently, polishing was performed on the cut samples to remove the burrs, surface unevenness and excess powder particles.

Post-process heat treatment of the prepared samples was conducted with the use of a furnace (Elite Thermal Systems Limited, BRF14/5-2416, Leicestershire, UK). The furnace was first heated up to a temperature that was 10 °C higher than the desired heat treatment temperature. The furnace was then opened up and its temperature was allowed to drop to the designated temperature. Following, the samples were sent into the furnace. Successively, the furnace was closed and its temperature was monitored. A timer of 5 min would be activated once the furnace temperature has reached the desired temperature. Subsequently, the samples were removed and air-cooled under atmospheric condition. The heat treatment was performed at four different temperatures, ranging from 400 to 700 °C, with an interval of 100 °C. After the heat treatment process, the samples were then further cooled down to a temperature that was below their M_f_ temperature. This ensured that the samples only exhibited twinned martensitic phase before any testing. Eight smaller samples and four long strip samples for each heat treatment temperature were produced to check for their repeatability.

### 2.3. Material Characterisation

Four properties of the repetitively scanned NiTi samples were determined: stress-free shape memory responses, phase transformation characteristics, phase analysis and the grain size.

The stress-free shape memory behavior of the long strip samples was determined under the tensile mode. The samples were first loaded up to a 6% strain, followed by unloading to a load of about 0.5 N (Shimadzu, AG–X Plus, Kyoto, Japan). A strain rate of 0.01%/min and room temperature condition were used for both loading and unloading processes. The 0.5 N load was then maintained constant throughout the remaining test to simulate a stress-free condition. Following, the samples were heated up with the use of a thermal chamber (Shimadzu, TCE-N300-CE, Kyoto, Japan) at a rate of about 2 °C/min. The magnitude of strain recovery due to shape memory effect was recorded. Elongation and thermal expansion of the clamps during the loading and heating processes were accounted for to ensure the high accuracy of the data collected. During the loading stage, an alumina plate was loaded from 0 to 200 N at room temperature to determine the clamps’ elongation. Its thermal expansion was obtained by subjecting the plate with a constant tensile load of 0.5 N and subsequent heating of the clamps from room temperature to 120 °C. The plate was presumed to has negligible deformation during both processes and the data recorded were mainly attributed to the elongation and thermal expansion of the clamps. A more accurate estimation of the samples’ deformation was gained by eliminating these data from the raw results collected during the respective loading and heating processes.

The transformation characteristics of the NiTi powder, non-heat treated (NT) and heat treated samples were determined by using the DSC (TA Instruments, DSC 2920 Modulated DSC, New Castle, Australia) equipment. The samples were subjected to five thermal cycles with a ramp rate of 5 °C/min from a range of −30 to 120 °C. Only the fifth cycle was used and presented in determining their phase transformation temperatures.

In order to investigate the phases present in the samples before and after the heat treatment, X-ray diffraction (XRD) (Malvern Panalytical Ltd., Empyrean, Royston, UK) was performed on the 5 mm by 10 mm samples with Cu-Kα radiation (wavelength = 1.5406 Å). The NiTi powder was also tested for comparison. Additionally, the integrated area under each diffraction peak for every sample was determined. The volume fraction of the phases present was estimated as the ratio of the peak areas [40,44,45,46].

The average grain size of the samples was measured according to the methods mentioned in ASTM E112-13 [47]. Both NT and heat treated 5 mm by 10 mm samples were hot mounted (Metkon Instruments Inc., Ecopress 100, Jakarta, Indonesia) into a polymer resin. These mounted samples were then subjected to polishing with diamond lapping films of different grades, starting from the coarsest of 18 µm and ending with the finest of 0.1 µm. After which, the polished samples were etched with Kroll’s Reagent (Best Chemical Co (S) Pte Ltd., 2–6% HNO_3_, 1–3% HF and 91–97% H_2_O, Singapore) for a period of 2 min. Lastly, the microstructures of the polished and etched samples were viewed using an optical microscope (Carl Zeiss, Axioskop 2 Mat, Baden-Württemberg, Germany) at a magnification of 50 times. Abrams three-circle procedure was utilised to determine the average grain size. The three-circle pattern was applied blindly to five different fields and the number of intersections was recorded. The ASTM grain size number and average grain size for each heat treatment condition were then determined.

## 3. Results

### 3.1. Shape Memory Responses

The main objective of introducing post-process heat treatment is to increase the transformation strain of the repetitively scanned SLM NiTi samples. Table 2 presents the average strain readings recorded during the tensile test for the NT samples, samples heat treated at 400 °C (H400), 500 °C (H500), 600 °C (H600) and 700 °C (H700). This temperature range was selected to include a 100 °C deviation from the recrystallization temperature (550 to 600 °C) of NiTi [48,49].

According to Table 2, H400 samples demonstrated an improvement in the transformation strain and shape recovery percentage as compared to the NT samples. However, a further increase in the heat treatment temperature resulted in a decreasing trend for both shape recovery and transformation strain.

### 3.2. Phase Transformation Characteristics

**Figure 2:**
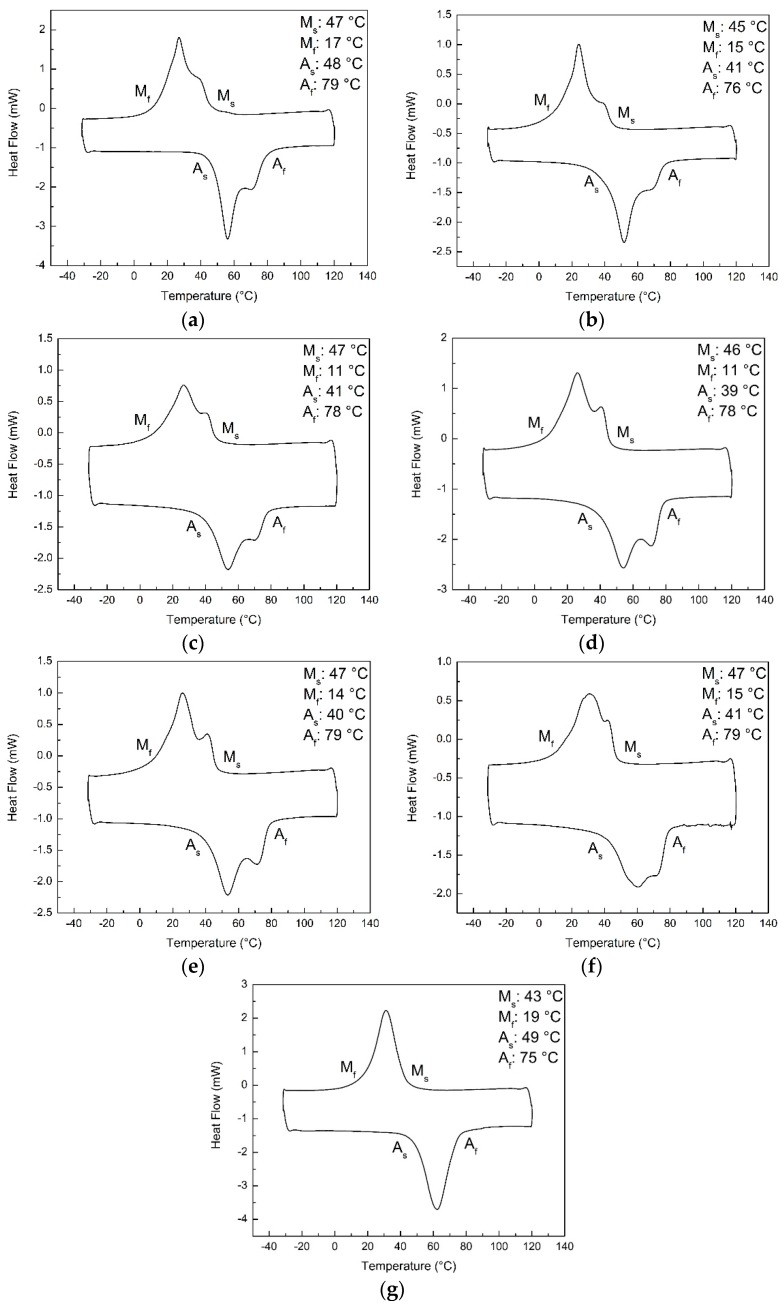
Fifth cycle of the differential scanning calorimetry testing of the (**a**) NiTi powder, (**b**) NT, (**c**) H400, (**d**) H500, (**e**) H600, (**f**) H700 and (**g**) conventionally optimised single scanned samples.

Figure 2 shows the fifth DSC cycles of NiTi powder, NT and heat treated samples. The DSC curve of the conventionally optimised single scanned sample is also shown for comparison. The single scanned sample was observed to demonstrate a single peak while the other samples exhibit double peaks during the heating and cooling processes. Table 3 reports their average transformation temperatures. The average transformation temperatures of the SLM NiTi materials before and after heat treatment were found to be similar to each other. However, the M_f_ temperature was observed to show a decreasing trend with an increase in the standard deviation, as the temperature of the heat treatment decreases. This could indicate some changes in the chemical composition of the samples as the heat treatment condition varies. This is due to the high dependency of the transformation temperatures on the chemical composition of NiTi [41,51,52].

### 3.3. Phase Analysis

**Figure 3:**
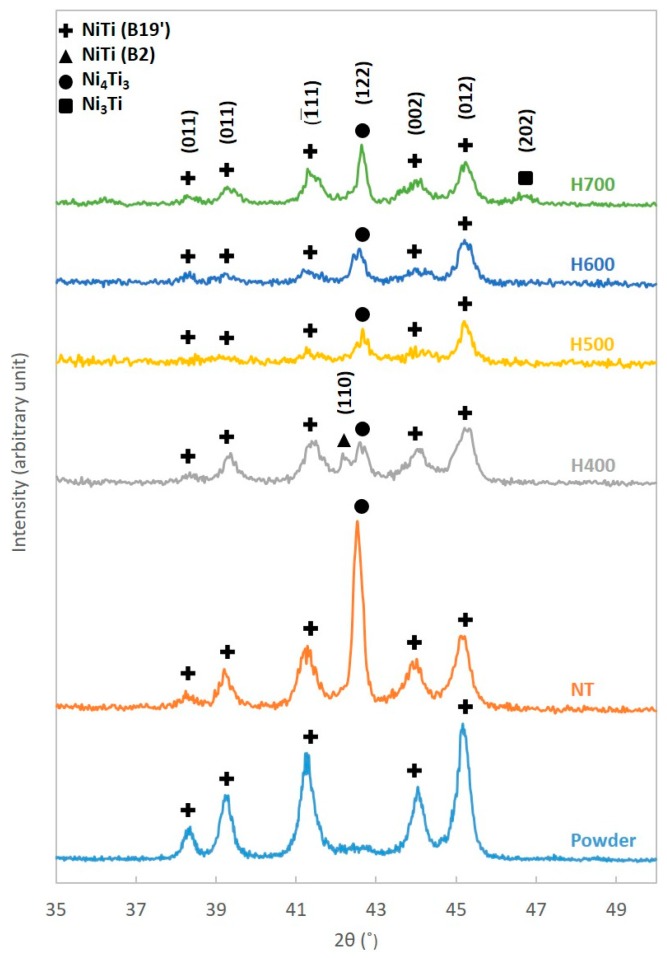
XRD patterns of NiTi powder, NT, H400, H500, H600 and H700 samples.

Due to the rapid solidification nature of the SLM process, there is a possibility of generating an internal stress field within the samples fabricated [2]. The stress field produced could have an adverse influence on the detwinning process [53]. Thus, heat treatment was introduced to eliminate the possible presence of the stress field. Nonetheless, heat treatment could also lead to the formation of precipitates, which might contribute to the better shape recovery observed [54]. The XRD patterns of the powder, NT and heat treated samples are presented in Figure 3.

In general, the XRD patterns of the powder and samples were similar. However, both NT and heat treated samples have demonstrated a Ni_4_Ti_3_ peak that was absent from the NiTi powder. Moreover, an additional austenitic peak NiTi (B2) (110) has also been observed in the XRD pattern of the H400 sample. The H700 sample has exhibited a Ni_3_Ti peak that was not seen in the XRD patterns of the other samples as well.

Meanwhile, Table 4 shows the estimated volume fraction of each phase present in the samples. Interestingly, the volume fraction of the martensitic phase increases with the implementation of heat treatment. The highest volume fraction of martensite could be found in the H400 samples. However, the concentration was observed to decrease sharply when the heat treatment temperature rises to 500 °C. Any further increment of the temperature has resulted in the slight fluctuation of the martensitic phase content.

### 3.4. Grain Size Measurement

**Figure 4:**
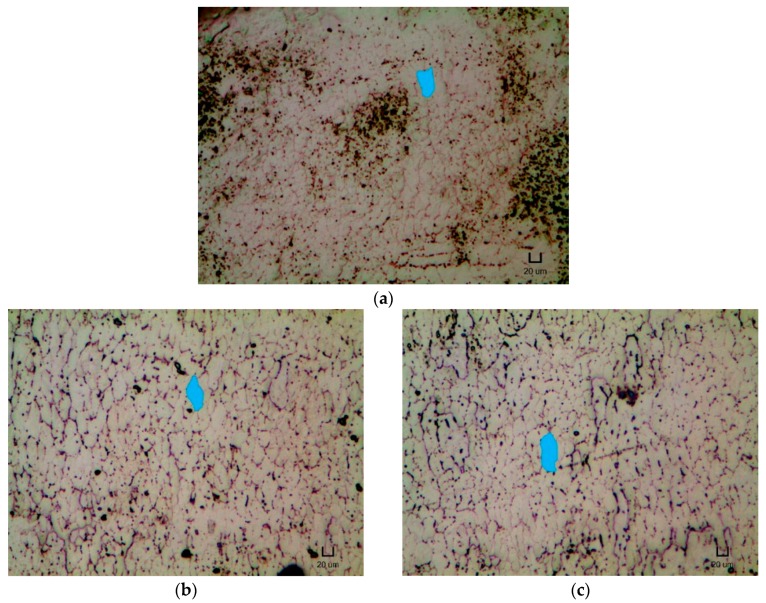

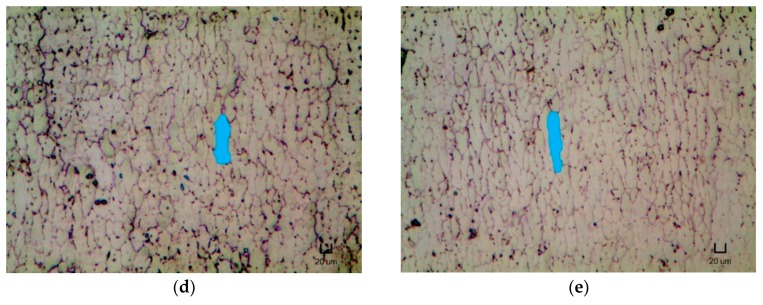
Micrographs of (**a**) NT, (**b**) H400, (**c**) H500, (**d**) H600 and (**e**) H700 samples under 50 times magnification with their typical grain shape highlighted. Scale bar: 20 µm.

Figure 4 presents the micrographs of the NT and heat treated samples. Table 5 shows the ASTM grain size number and average grain area for each heat treatment condition. It is to be noted that a higher ASTM grain size magnitude implies a larger amount of grains per unit area (or a smaller grain size).

From Table 5, the ASTM grain size number and average grain area are observed to decrease and increase, respectively, with the implementation of heat treatment and with increasing heat treatment temperature. This result also tallies with the micrographs shown in Figure 4, where the typical shape of the grain is highlighted. The grains appeared to experience grain growth and elongate lengthwise upon the heat treatment with increasing temperature.

## 4. Discussion

### 4.1. Analysis of Phase Transformation Characteristics

The formation of the rhombohedral phase (R-phase) was reported to be a result of Ni_4_Ti_3_ precipitation [22,30,55,56]. Initially, it was believed that the R-phase causes the double peaks found in the DSC curves of the NiTi powder, NT, H400 and H500 samples [11,22,30,41,49,57]. This impression was supported by their XRD patterns, where the presence of Ni_4_Ti_3_ has been detected [22,30,55,56]. Nevertheless, studies have shown that heat treatment conducted at 600 °C and above would lead to the disappearance of the R-phase [49,58]. However, double peaks are still observable in the DSC curves of the H600 and H700 samples as seen in Figure 2e,f. Moreover, the transformation temperatures of the R-phase and martensitic phase tends to be separable [35]. The additional peak would be divided and distinct from the martensitic peak. Conversely, the exhibited double peaks from NT and heat treated samples appeared close and overlapped each other. Thus, it can be concluded that the origin of the double peaks is not due to the formation of Ni_4_Ti_3_ precipitates.

It is hypothesized that the proximity of the two peaks is caused by the chemical inhomogeneity of the NiTi powder and repetitively scanned samples. The average atomic percentage of the powder was determined to be 50.16% Ni and 49.84% Ti. According to the NiTi phase diagram, the boundary of the Ni-rich region decreases significantly with decreasing temperature [35]. Furthermore, the solubility of NiTi becomes negligible when its temperature drops to below 600 °C. Therefore, the material would be in a metastable equilibrium state where both NiTi and Ni_4_Ti_3_ precipitates would coexist in equilibrium.

However, one of the objectives of introducing repetitive scanning is to control the amount of energy absorbed by the NiTi material during the second scan [31]. The formation of the through cavities is eliminated when less energy was absorbed by the partially melted NiTi during the second scan, as compared to energy absorbed by the powder during the conventional single scan process. Hence, the implementation of repetitive scanning lowered the temperature of the molten NiTi. Thus, it resulted in a shorter time to complete the solidification process.

Similarly, during the gas atomization of the NiTi ingot, the material experienced a high solidification rate and a short solidification time. As a result, the atoms would have little time to diffuse. Therefore, the formation of the Ni_4_Ti_3_ precipitates was suppressed [35,51]. Consequently, it could lead to two peaks observed in the DSC curves, where one peak corresponds to the NiTi matrix, while the other peak corresponds to the slightly Ni-rich transition region. This slight compositional segregation is caused by the incomplete diffusion of the atoms to form Ni_4_Ti_3_ precipitates.

This hypothesis is also supported by the phase transformation characteristics of the single scanned sample as shown in Figure 2g. To achieve full melting of the powder particles in a single scan, the amount of linear energy density directed to the powder bed would be relatively high. However, the high laser absorptivity and low heat conductivity of the powder particles contributed to the huge amount of energy absorbed by the material [33]. Thus, the temperature of the molten pool would reach a higher magnitude, resulting in more time required to dissipate the heat and solidify. Subsequently, the atoms would have sufficient time to diffuse and rearrange to obtain a more homogenous chemical composition, with or without the formation of precipitates. Hence, only a single peak was observed during the DSC testing of the conventionally optimised single scanned sample.

Nevertheless, the presence of the double peaks after repetitive scanning may not be a drawback. Instead, it encourages the implementation of repetitive scanning as double peaks can be found in the DSC curves of the NiTi powder as well. The conservation of the double peaks suggested the ability of the repetitive scanning approach to retain the properties of the raw material used. This is especially important since the SLM process involves both melting and solidification processes. One of the common problems encountered includes the alteration of the material’s properties after the SLM fabrication. For instance, huge deviations in the transformation temperatures due to Ni evaporation during the melting phase have been observed [9,11,20,25,26]. Therefore, in the future research, homogenous NiTi powder and repetitive scanning approach can be utilised together. It is expected that the repetitively scanned SLM NiTi parts will exhibit a single peak.

### 4.2. Analysis of Shape Memory Responses

#### 4.2.1. Formation and Effects of Ni_4_Ti_3_ Precipitates

Based on the XRD patterns, no traces of Ni_4_Ti_3_ precipitate can be found on the NiTi powder. However, the presence of this intermediate phase has been identified on the SLM NiTi samples. One possible explanation would be that during the fabrication procedure, the melting stage of the SLM process provided an additional opportunity for the atoms to complete diffusion. Thus, it resulted in the formation of Ni_4_Ti_3_ precipitates. No precipitation has been detected in the NiTi powder as the rapid solidification rate of the gas atomization process did not provide sufficient time for complete diffusion. Hence, only the transition region was observable in the DSC curve of the NiTi powder. Both the transition region and Ni_4_Ti_3_ precipitates found in the DSC curve and XRD pattern of the NT samples were due to the partial completion of the diffusion process.

Nevertheless, during the process of heat treatment, the occurrence of oxidation was inevitable. The manifestation of oxidation would lead to the depletion of Ti as it was more reactive to oxygen than Ni [59]. Thus, the content of Ni in the NiTi matrix would increase, resulting in two phenomena; (1) the decrease of the transformation temperatures and the transformation of martensitic phase into austenitic phase and (2) the promotion of precipitate formation.

Precipitation will occur when the condition of supersaturation is met. The NiTi material would be in a supersaturated state when the content of Ni has exceeded its solubility limit but yet to form precipitation due to the quenching process. Hence, the supersaturation state of NiTi would provide a driving force for the initiation of precipitation during the heat treatment process [35].

When the temperature of the heat treatment conducted was at 400 °C, the depletion of Ti would not be as much. This was because the main oxidation process did not start immediately at a lower temperature [59]. Moreover, the oxidation rate tended to slow down and saturate after some time. Thus, the slight increase in the Ni content could be within the overlapped region between the metastable equilibrium phase (NiTi and Ni_4_Ti_3_) and the solubility limit of NiTi [35,41]. In this region, the increase in the Ni content would lead to the decrease in the transformation temperatures. This decrease was reflected in the M_f_ temperature of the H400 samples while the identification of the austenitic phase was shown in their XRD pattern. Likewise, Firstov et al. have also reported the detection of austenitic phase when their NiTi material (atomic percentage of 50% Ni and 50% Ti) was processed between a heat treatment temperature of 300 to 500 °C [59]. Additionally, the small increase in the content of Ni would also result in a slight increase in the nucleation of the Ni_4_Ti_3_ precipitates.

However, when the heat treatment temperature increased to 500 and 600 °C, more Ti would be depleted. This is the result of oxidation proceeding earlier when at a higher temperature [59]. Thus, the increase in the content of Ni could exceed the solubility limit of NiTi, extending into the metastable equilibrium state. Subsequently, the austenitic phase disappeared as presented in the XRD patterns of the H500 and H600 samples. Instead, the rate of precipitation increased as shown in Table 4 and the existing Ni_4_Ti_3_ precipitates started to grow larger at the expense of the smaller particles [60]. The results obtained were also supported by the similar observation of Ni_4_Ti_3_ precipitates found in samples that were heat-treated at 500 to 600 °C for 6 min [35,61].

Nonetheless, when the heat treatment temperature increased to 700 °C, the depletion in Ti was so strong that the Ni_4_Ti_3_ precipitates began to decompose into the equilibrium Ni_3_Ti phase [35,56,59,62,63]. Hence, both Ni_4_Ti_3_ and Ni_3_Ti precipitates could be found in the XRD pattern of the H700 sample. The results obtained were confirmed by the observation of Ni_3_Ti precipitates at a higher heat treatment temperature of above 600 °C [59].

Conversely, a higher nucleation rate and the decomposition of Ni_4_Ti_3_ precipitates into Ni_3_Ti phase may not be beneficial to the shape memory properties of repetitively scanned NiTi samples. Based on Table 2, only H400 samples have demonstrated improvements in the transformation strain and shape recovery percentage as compared to the NT samples. The shape memory properties were found to deteriorate with increasing heat treatment temperature. The occurrence of these phenomena could be understood from the physical metallurgy principle.

The generation of a high density of fine precipitates is widely known to be the most effective method in preventing the movement of dislocations [35,56,64]. Concurrently, it increases the critical stress magnitude for the slip to occur as well [37]. The formation of dislocations is plastic deformation and an irreversible process. It is not possible to restore the strains produced by this defect via the mechanism of the reversible martensitic phase transformation. Therefore, heat treating the repetitively scanned NiTi samples at a temperature of 400 °C for a duration of 5 min would initiate the precipitations of fine Ni_4_Ti_3_ particles to deter the generation of plastic deformation. This result has also coincided with several past research where the conventionally-produced NiTi parts demonstrated the best SME and superelastic properties after heat treating at 400 °C [37,49,63]. Other studies have attributed the improvement in the shape memory properties to the presence of fine Ni_4_Ti_3_ precipitates as well [30,63,65,66].

Nevertheless, as the temperature of the heat treatment increases, particle agglomeration of the Ni_4_Ti_3_ precipitates occurs [30,48,62,65,66]. Experimental observation of Ni_4_Ti_3_ enlargement by 30 times as the heat treatment temperature raised from 400 to 500 °C has been reported by Yan et al. [63]. Thus, the increment of the heat treatment temperature would decrease the density distribution of the precipitates. It would then lead to a reduction of their effects on the shape memory properties of the heat treated NiTi samples. In addition, it has been reported that coarse Ni_4_Ti_3_ would lose its coherency with the NiTi matrix [48,62,63]. Hence, dislocations would be introduced to relieve the stress fields generated around the precipitates [58,62,63]. Consequently, it would lead to an overall decrease in the fatigue strength of the NiTi samples.

Meanwhile, Table 4 provides another explanation for the improvement in the shape memory properties exhibited by the H400 samples. It has identified that the highest volume fraction of martensitic phase could be found in the H400 samples. Thus, a larger transformation volume is available for the reversible martensitic phase transformation during the process of heating [40]. Correspondingly, the H400 samples would demonstrate a higher transformation strain than the other samples. In summary, the heat treatments of repetitively scanned NiTi samples above 400 °C have resulted in overaging of the material [64]. Eventually, the samples demonstrated poorer shape memory responses than the NT samples.

#### 4.2.2. Formation and Effects of Grain Boundary Migration

Other than introducing the formation of Ni_4_Ti_3_ metastable precipitates, the implementation of heat treatment was also found to alter the microstructures of the repetitively scanned NiTi samples. According to Figure 4 and Table 5, the grain size of the NT and heat treated samples increases with the application of heat treatment and with rising heat treatment temperature. Specifically, the grains were observed to elongate lengthwise.

At first glance, it might appear that the heat treated samples have undergone through the recovery and recrystallisation processes. The migration of the grain boundary was the result of ordinary grain growth after recrystallisation as the grains grew larger at the expense of the other grains. However, on closer inspection, it is inferred that the heat treated samples did not experience recrystallisation. This deduction came about based on the following factors.

For the recrystallisation process to occur, there are a few requirements that need to be fulfilled. Firstly, the samples have to be subjected to a certain magnitude of deformation [67]. It has been reported that the recrystallisation phenomenon did not happen for samples deformed below the strain of 20% [68]. Moreover, the recrystallisation temperature is a function of the degree of deformation [67]. A less deformed sample would have a higher recrystallisation temperature than a severely deformed sample. As reported in the past research, NiTi has a recrystallisation temperature of between 550 to 600 °C when they experienced cold-working of about 30% [48,49]. During the sample preparation, precautions were taken to ensure that the samples did not suffer any unnecessary deformation prior to the heat treatment and the determination of grain size. Thus, it is expected that the heat treated samples would not experience recrystallization within the implemented temperature range.

The second factor is the lack of time for the formation and growth of the recrystallised nuclei to microscopic size [69,70]. During a typical recrystallisation process, an initial incubation period is required for sufficient energy to develop such that the first strain-free nucleus could grow to a visible size [67]. In addition, the recrystallisation temperature for a particular material denotes the approximated temperature at which its highly cold-worked form would completely recrystallise in 1 h [67,70]. A heat treatment of 5 min is unlikely able to accumulate an adequate amount of energy to form defect-free nuclei and grow to appreciable size.

The third condition is the additional energy required to initiate the formation of the strain-free lattice [67]. During the process of cold-working, plastic deformation was introduced into the samples. The internal energy evolved from the deformation would increase the energy state of the atoms. However, it is not possible for the atoms or dislocations to revert back to a defect-free lattice from the distorted lattice at room temperature due to the nature of strain hardening. Hence, additional energy is required to bring the atoms to the next energy state level to overcome the rigidity of the distorted lattice. The additional amount of energy would be supplied in terms of heat energy. Nonetheless, as the repetitively scanned samples were not severely deformed prior to the heat treatment, the internal energy state of the samples would be much lower. Thus, a larger amount of additional energy needs to be provided to the samples to initiate recrystallisation. Since the highest heat treatment temperature tested is only about 100 °C higher than the recrystallisation temperature of a heavily deformed NiTi material, the energy supplied may not be sufficient to start up the recrystallisation process.

Besides needing to fulfil the three requirements, the observed grain shape also provided evidence of the absence of recrystallisation. In Figure 4, the grains were noticed to elongate upon heat treatment and with increasing heat treatment temperature. Moreover, the elongation seems to start promptly at the start of the heat treatment despite the short duration of 5 min. However, in a typical recrystallisation process, no observable differences can be seen in the microstructures of the material during the recovery phase [67]. Even when the recrystallisation temperature has been reached, it requires a certain period of incubation for the formation and growth of new nuclei. Furthermore, the recrystallised grains were observed to be equiaxed [67], which contradicted what was captured on the microscopic images. Thus, it is concluded that the heat treated samples did not experience recrystallisation. The elongation of the grains is postulated to be a result of strain-induced boundary migration [69].

The difference between strain-induced boundary migration and the recrystallisation process is that in the former, the annealed material left behind due to the movement of the grain boundary has the same orientation as the strain hardened parent grain [69]. However, in the recrystallisation process, nuclei with different orientations are produced. The movement of the grain boundaries in the strain-induced boundary migration is towards the distorted regions due to the strain gradients generated [71]. Correspondingly, this would result in the increase of the size of one grain and the disappearance of the other grain. One characteristic of strain-induced boundary migration is that the produced annealed material would be constricted by the ordinary grain boundary on one side, while bounded by the parent grain on the other side without the intervention of grain boundary [69]. Nevertheless, in a typical recrystallisation process, the produced recrystallised grains would be located in between the strain hardened grains. These grains were separated by grain boundaries.

**Figure 5:**
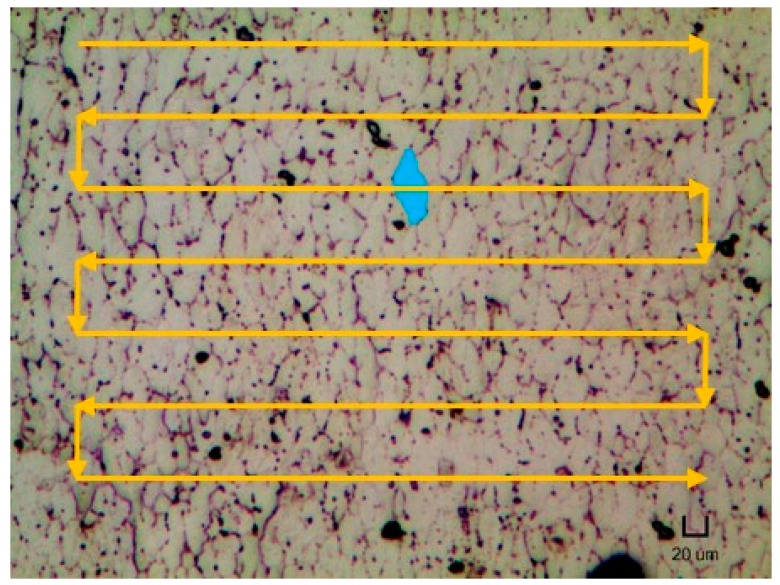
Micrograph of the H400 sample with its typical grain shape highlighted and with the schematic of laser scanning strategy.

However, the reason for the occurrence of the strain-induced boundary migration on repetitively scanned samples is not known yet at this moment. Nonetheless, the grains were observed to elongate together in one direction. The direction of the grain elongation was generally aligned perpendicularly to the direction of the laser scanning path as illustrated in Figure 5. One possible explanation for both phenomena would be due to the directional solidification nature of the SLM process. During the fabrication of a single layer, the laser would begin scanning in a line-by-line manner, starting from the top region. As the laser proceeds down to the adjacent line, the previous scanned section could have solidified and cooled down. Hence, a temperature gradient is generated between the molten and solidified regions, resulting in the grains to grow towards the direction of the temperature gradient. In addition, the non-uniform thermal conductivity and the thermal expansion coefficient of the liquid and solid phase of NiTi could also lead to the development of residual stress and/or strain. Thus, during the heat treatment process, the grains further elongate lengthwise according to the produced strain gradients. Nevertheless, more studies are needed to confirm this hypothesis.

In general, the occurrence of the strain-induced boundary migration did not contribute to the improvement in the shape memory properties of repetitively scanned NiTi samples. As presented in Figure 4, Table 2 and Table 5 the transformation strain and shape recovery percentage of the heat treated samples decreased with increasing grain size. The only exception is the H400 samples, where they demonstrated an improvement in their shape memory responses as compared to the NT samples. However, this enhancement could be attributed to the formation of fine Ni_4_Ti_3_ precipitates and high content of martensitic phase, where they outweigh the negative impact of the strain-induced boundary migration.

As reported by various authors, the increase in grain size could be detrimental to the mechanical properties of repetitively scanned NiTi samples [36,48,72]. Based on the results obtained by Delville et al., they observed that samples with a larger grain size tend to be significantly prone to the formation and build-up of dislocations [72]. The increase in the dislocation density has led to the rapid accumulation of permanent strain build-up during the cyclic test performed. Moreover, they determined that their NiTi samples had the highest resistance to slip deformation when the samples just entered into the recrystallisation phase. In this phase, the new defect-free recrystallised grains would be coexisting with the polygonised microstructure. Furthermore, the exhibition of small grain size contributed to its high strength and stability for the cyclic test as well. However, when the heat treatment process proceeded into the phase of grain growth, the yield stress for plasticity and strength dropped significantly.

The improvement in the mechanical properties of NiTi SMA after a decrease in the grain size has also been presented by the other researchers [36,48]. It was found that a reduction in grain size could slow down the propagation of cracks formed during the cyclic test of NiTi material. Hence, samples with small grains would have a higher fatigue life than samples with coarse grains. The reason for the improvement in the NiTi shape memory properties is due to the increase in the volume fraction of the grain boundary [36]. The grain boundary acts as a non-transformable barrier that separates the crystallites with different orientations. When the grain size decreases, the mechanical constraint of the grain boundary on the deformation of the crystallites becomes more significant as compared to samples with coarse grains. Thus, the movement of the dislocation was impeded, resulting in a decrease in the accumulation of permanent strain. Sequentially, samples with finer grain size would have a higher transformation strain and shape recovery percentage. Therefore, combining the effects of grain size and Ni_4_Ti_3_ precipitates, H400 samples have the best shape memory properties among the other heat treated samples.

## 5. Conclusions

**Figure 6:**
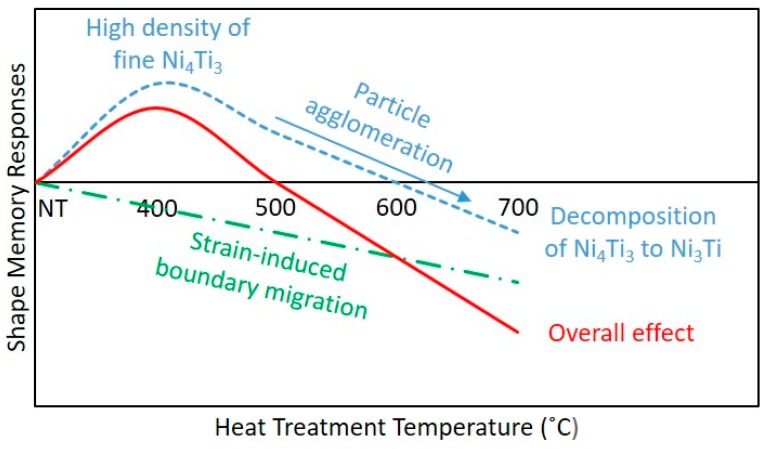
Schematic of the overall effects of heat treatment on the shape memory responses of repetitively scanned NiTi samples.

In the prior research, repetitively scanned NiTi samples have demonstrated the ability to be tensile loaded. They exhibited an average of 4.61% transformation strain. Nonetheless, there is still room for improvement when being compared to the 6% transformation strain demonstrated by the conventionally-produced NiTi parts. Hence, in this paper, post-process heat treatment was implemented to enhance the shape memory properties of the repetitively scanned NiTi samples. The overall effects of the heat treatment process are summarised in Table 6 and Figure 6.

As the heat treatment implemented could have both positive and negative influences, it is necessary to achieve a balance between them. The results obtained showed that heat treating the repetitively scanned samples at a temperature of 400 °C for a duration of 5 min has led to an improvement in their transformation strain and shape recovery percentage. With the use of this heat treatment condition, a high density of fine Ni_4_Ti_3_ metastable phase was produced. Concurrently, the samples had the highest volume fraction of martensitic phase as well. Nonetheless, strain-induced grain boundary migration was observed. Even though the increase in the grain size has detrimental effects on the mechanical properties of NiTi SMA, the positive impacts of Ni_4_Ti_3_ precipitate formation and the high content of martensitic phase outweigh the negative influences as illustrated in Figure 6. A high density of these fine precipitates has effectively increased the critical stress level for plastic deformation to occur and successfully impede the movement of dislocations during tensile testing. The high concentration of martensitic phase has allowed a larger volume of phase transformation to take place during the deformation and shape recovery processes. Therefore, the H400 samples were able to demonstrate an improvement in their shape memory properties as compared to the NT samples. Nevertheless, heat treating the repetitively scanned samples above this temperature has resulted in over-aging (agglomeration of Ni_4_Ti_3_, reduction in the martensitic phase and further increment of grain size). As a consequence, degradations in the shape memory properties were inevitable. Hence, for the future applications, it is recommended not to heat treat the repetitively scanned NiTi samples above 400 °C.

## Figures and Tables

**Table 1:** Chemical composition of NiTi powder based on EDX results.

Scanned Section	Atomic Percentage of Ni (%)	Atomic Percentage of Ti (%)
1	51.07	48.93
2	48.99	51.01
3	51.19	48.81
4	48.30	51.70
5	49.74	50.26
6	51.24	48.76
Average	50.09	49.91

**Table 2:** Average strain readings for both non-heat treated and heat treated repetitively scanned Selective Laser Melted NiTi samples tested under the tensile mode [50].

Strains/Samples	NT	H400	H500	H600	H700
Residual Strain (%)	5.14 ± 0.11	4.99 ± 0.01	4.96 ± 0.09	5.01 ± 0.07	4.96 ± 0.02
Transformation Strain (%)	3.37 ± 0.19	3.59 ± 0.17	3.36 ± 0.28	3.18 ± 0.21	3.08 ± 0.30
Shape Recovery = $\frac{\mathrm{Transformation}\;\mathrm{Strain}}{\mathrm{Residual}\;\mathrm{Strain}}$ (%)	0.66 ± 0.04	0.72 ± 0.04	0.68 ± 0.05	0.63 ± 0.04	0.62 ± 0.06

**Table 3:** Average transformation temperatures of the NiTi powder, NT and heat treated samples.

Samples/Transformation Temperatures	M_s_ (°C)	M_f_ (°C)	A_s_ (°C)	A_f_ (°C)	(A_f_ − A_s_) (°C)
NiTi Powder	47 ± 0.0	17 ± 0.0	48 ± 0.7	79 ± 0.0	31 ± 0.7
NT Samples	45 ± 0.0	15 ± 0.8	40 ± 1.1	76 ± 0.0	36 ± 1.1
H400 Samples	47 ± 0.5	12 ± 2.9	40 ± 1.3	78 ± 0.7	38 ± 0.8
H500 Samples	47 ± 0.5	11 ± 2.2	41 ± 1.2	79 ± 0.5	38 ± 0.9
H600 Samples	47 ± 0.0	14 ± 1.1	40 ± 1.2	79 ± 0.0	39 ± 1.2
H700 Samples	48 ± 0.5	15 ± 1.1	41 ± 1.1	79 ± 0.4	38 ± 1.2

**Table 4:** Estimated volume fraction of the phases present in the NiTi powder, NT and heat treated samples.

Samples/Phases	Martensitic Phase (%)	Austenitic Phase (%)	Ni_4_Ti_3_ (%)	Ni_3_Ti (%)
NiTi Powder	100.0	-	-	-
NT Sample	64.4	-	35.6	-
H400 Sample	89.4	2.4	8.2	-
H500 Sample	72.3	-	27.7	-
H600 Sample	77.8	-	22.2	-
H700 Sample	74.0	-	19.0	7.0

**Table 5:** Average ASTM grain size number and grain area for both NT and heat treated samples.

Parameters/Samples	NT	H400	H500	H600	H700
ASTM Grain Size Number	3.35 ± 0.07	2.97 ± 0.07	2.37 ± 0.14	2.05 ± 0.18	1.74 ± 0.12
Average Grain Area (μm^2^)	12,830 ± 698	16,634 ± 897	25,296 ± 2632	31,696 ± 4007	39,173 ± 3396

**Table 6:** Overall effects of heat treatment on repetitively scanned NiTi samples.

Positive Impacts	Negative Impacts
Precipitation of a high density of fine Ni_4_Ti_3_ precipitates at a lower heat treatment temperature of 400 °C	Initiation of particle agglomeration and formation of dislocations with increasing heat treatment temperature from 500 to 700 °C
Improvements in the transformation strain (from 3.37 ± 0.19 to 3.59 ± 0.17) and shape recovery percentage (from 0.66 ± 0.04 to 0.72 ± 0.04) of repetitively scanned NiTi samples	Occurrence of strain-induced boundary migration with the implementation of heat treatment and with rising heat treatment temperature from 400 to 700 °C
